# Draft Genome Sequence of *Nonlabens ulvanivorans*, an Ulvan-Degrading Bacterium

**DOI:** 10.1128/genomeA.00793-14

**Published:** 2014-08-14

**Authors:** Moran Kopel, William Helbert, Bernard Henrissat, Tirza Doniger, Ehud Banin

**Affiliations:** a Institute for Nanotechnology and Advanced Materials, Bar-Ilan University, Ramat Gan, Israel; d Mina and Everard Goodman Faculty of Life Sciences, Bar-Ilan University, Ramat Gan, Israel; b Centre de Recherches sur les Macromolécules Végétales, Université Joseph Fourier and Institut de Chimie Moléculaire de Grenoble, Grenoble, France; c Architecture et Fonction des Macromolécules Biologiques, Centre National de la Recherche Scientiﬁque & Aix-Marseille University, Marseille, France

## Abstract

Here we report the draft genome sequence of the bacterium *Nonlabens ulvanivorans*, which was recently isolated. To our knowledge, this is the first published genome of a characterized ulvan-degrading bacterium. Revealing the ulvan utilization pathways may provide access to a vast marine biomass source that has yet to be exploited.

## GENOME ANNOUNCEMENT

*Ulvales* (*Chlorophyta*) are worldwide-distributed green seaweeds (1). Species of the genus *Ulva* are the main contributors to green tides, which have become an increasing economic and environmental problem (2). Although the majority of biomass washed ashore is of little use, the algae’s dry weight is constituted of up to 54% carbohydrates (1). The main carbohydrate portion is attributed to the sulfated polysaccharide ulvan, embedded in the algal cell wall (3). Ulvan composition and structure have been studied in depth for its varied polymeric properties and biological activities (1, 3). l-Rhamnose 3-sulfate, d-glucuronic acid, l-iduronic acid, and d-xylose are the main polymer building blocks, conventionally distributed along the polymer as disaccharides (ulvanobiuronic acid type A and B) (3).

*Nonlabens ulvanivorans* was isolated from the feces of the sea slug *Aplysia punctata*, which feeds on *Ulva* (4). A novel ulvan lyase was purified from an *N. ulvanivorans* batch culture, sequenced, and heterologously overexpressed in *Escherichia coli*, and its ability to depolymerize ulvan was characterized (5). Unsaturated β-glucuronyl hydrolase, an ulvan-cleaving enzyme that digests the particular oligosaccharides produced by the ulvan lyase, was found in the vicinity of the ulvan lyase on the *N. ulvanivorans* genome (6). The other enzymes required for complete saccharification of the ulvan polysaccharide remain unidentified.

*De novo* sequencing of the ulvan-degrading *N. ulvanivorans*, was conducted by the Technion LS & E. Infrastructure Unit, Israel. DNA libraries were generated using an Illumina TruSeq DNA kit. The libraries were diluted to 2 nM and run on an Illumina HiSeq 2000 at a final concentration of 8 pM in 100-bp (×2) paired-end sequencing. Four samples were loaded into one lane to receive 58 million passed filter reads for each sample. Data were analyzed using the *de-novo* assemblers AbySS (7) and Velvet (8). The final draft comprises 45 contigs (longer than 200 bp), with a mean size of 720,575 bp and a maximum length of 1,660,217 bp. The total length of the genome was 3,220,243 bp with a mean GC content of 35% and an average coverage of approximately 1,933×. Contig annotation was conducted using the Rapid Annotations using Subsystems Technology (RAST) server (9). The final draft contains 2,954 coding sequences (of which 1,752 possess annotated functions and 1,202 are hypothetical proteins), 21 tRNA genes, and 1 rRNA operon. The number of rRNA operons is consistent with the number of rRNA copies estimated by the Ribosomal RNA Database (rrnDB) for the family *Flavobacteriaceae* (between 1 and 6 copies) (10). A plasmid-partitioning gene, parA, was detected on contig 98, which suggests the occurrence of a plasmid.

The first draft genome sequence of an ulvan-degrading bacterium provides a major step toward the unveiling of other enzymes that participate in *Ulva* degradation and *Ulvales* cell wall polysaccharide utilization. Furthermore, it may allow for better characterization and even modification of these untapped polysaccharides in the near future.

### Nucleotide sequence accession numbers.

This whole-genome shotgun project has been deposited at DDBJ/EMBL/GenBank under the accession number JPJI00000000. The version described in this paper is version JPJI01000000
